# Continued confusion in mild traumatic brain injury hyperbaric oxygen studies

**DOI:** 10.1016/j.conctc.2024.101348

**Published:** 2024-08-12

**Authors:** Paul G. Harch

**Affiliations:** Clinical Professor of Medicine, Department of Medicine, Section of Emergency Medicine, Hyperbaric Medicine, Louisiana State University Health Sciences Center, New Orleans, LA, USA

Dear Sirs:

Wright et al. [1] in their proposed study of hyperbaric oxygen therapy (HBOT) in mild traumatic brain injury/postconcussion syndrome (mTBI/PCS), state that the evidence for HBOT in PCS “… is mixed due to inconsistencies in the treatment protocol and the focus on veterans with combat injuries ….” In fact, the evidence is consistent when assessed scientifically by dose [2]; it is the design of the studies and the interpretation of the data that is inconsistent [2]. The crucial flaw in these studies is the incorporation of control groups based on the traditional unscientific definition of HBOT [3] that omits barometric pressure [4]. HBOT and hyperbaric therapy are composed of at least two bioactive components, increased barometric pressure and pressure of oxygen [5] with a potential third component, “inert” gas pressure, when the FiO2 is less than 100 % [6]. The authors acknowledge this third component on page 2, end of first full paragraph. Control/sham groups have to control for each of these variables. Wright et al. [1] and all of the military studies [[7], [8], [9], [10]] do not control for pressure and Wright et al. [1] and Cifu et al.’s [7] control groups add the third uncontrolled component, “inert” gas pressure.

The authors aim to “expand upon the existing research” with a “well-designed” study that accounts “for the limitations of the prior research ….” Unfortunately, their design has serious limitations and is going to perpetuate and augment the confusion and controversy. The biggest problem in their study is the sham/control group which only controls for the oxygen component [2,[4], [5], [6]]. This is duplicating the same flawed control group in Cifu et al. [7] (refs.15-17, 21 in Wright et al. [1]). Wright et al. [1] are also not using “… a novel placebo gas system …” This identical novel system was utilized in Cifu et al. [7] and produced insignificant results for mTBI/PPCS (persistent postconcussion syndrome), while disproving the “ritual” placebo effect used by the military's researchers to explain positive data in the other studies' control groups [8]. Cifu et al. [7] used 40 treatments for 60 minutes each, instead of Wright et al.’s [1] proposed 20 treatments for 90 minutes each, but Wright et al. [1] claims to use it in a different subject group, the “subacutely” injured, 3–12 months post-injury. While definitions of subacute differ, the condition they are treating, PPCS, is defined as symptom persistence for 3 months or greater and is considered chronic [11]. Essentially, their subject group will be identified with the previous TBI/HBOT studies [[7], [8], [9], [10]], the 3–12 month inclusion criterion will overlap with two of the studies [7,9], the study will use a number of HBOTs that are historically insufficient to treat chronic conditions [12], and it will involve another non-sham non-control control group. With this design, expansion of the research and accounting for previous limitations is highly unlikely.

In the interest of achieving the aims of Wright et al. [1] it is humbly suggested that the authors reconsider their protocol: omit the use of the word sham, add a non-treatment control group [8], and increase the number of HBOTs/subject to at least 30.

## Declaration of competing interest

The authors declare the following financial interests/personal relationships which may be considered as potential competing interests:

The author is the owner of an S Corporation that is the vehicle for his practice of hyperbaric medicine.
